# Genipin modified lyophilized platelet-rich fibrin scaffold for sustained release of growth factors to promote bone regeneration

**DOI:** 10.3389/fphys.2022.1007692

**Published:** 2022-09-30

**Authors:** Xiaoyao Liu, Mingjing Yin, Ying Li, Jianqun Wang, Junlong Da, Zhongshuang Liu, Kai Zhang, Lixue Liu, Wenxuan Zhang, Peijun Wang, Han Jin, Bin Zhang

**Affiliations:** ^1^ Heilongjiang Provincial Key Laboratory of Hard Tissue Development and Regeneration, The Second Affiliated Hospital of Harbin Medical University, Harbin, China; ^2^ Department of Stomatology, The First Affiliated Hospital of Harbin Medical University, Harbin, China; ^3^ Department of Stomatology, Shenzhen University General Hospital, Shenzhen University, Shenzhen, China; ^4^ Heilongjiang Academy of Medical Sciences, Harbin, China

**Keywords:** lyophilized PRF, genipin, sustained release, growth factor, bone tissue engineering

## Abstract

Lyophilized platelet-rich fibrin (L-PRF) was shown to further activate resident platelets in platelet-rich fibrin causing a higher amount of growth factors release. However, it still required further experimental studies to resolve the uncontrolled degradation and burst release problem. In this study, the nature crosslinker genipin is introduced to improve the performance of L-PRF scaffold. We used a series of gradient concentration genipin solutions to react with L-PRF. The crosslinking degree, micro morphology, mean pore size, water absorption and mechanical properties of the crosslinked scaffold were evaluated. In order to study the effect of genipin modification on the release kinetics of growth factors from L-PRF, we detected the release of platelet-derived growth factor, vascular endothelial growth factor and transforming growth factor *in vitro* by ELISA. To investigate the biodegradability of the crosslinked L-PRF *in vivo*, the scaffolds were transplanted subcutaneously into backs of rats, and the materials were recovered at 1, 2 and 4 weeks after implantation. The biodegradation, inflammatory reaction and biocompatibility of the scaffolds were examined by histological staining. Finally, the genipin crosslinked/uncrosslinked L- Platelet-rich fibrin scaffolds were implanted with freshly prepared SHED cell sheets into rat critical size calvarial defects and the skull samples were recovered to examine the treatment efficacy of genipin crosslinked L-PRF by histologic and radiographic approaches. Results of this study indicated that genipin can be used to modify L-PRF at room temperature at a very low concentration. Genipin-modified L-PRF shows better biomechanical performance, slower biodegradation, good bioavailable and sustained release of growth factors. The 0.01% w/v and 0.1% w/v genipin crosslinked L-PRF have good porous structure and significantly promote cell proliferation and enhance the expression of key genes in osteogenesis *in vitro*, and work best in promoting bone regeneration *in vivo*.

## Introduction

Bone tissue defects caused by periodontal disease, periapical disease, trauma and tumor that typically lead to inadequate bone volume are still challenges for dentists (Stumbras et al., 2019). Bone healing is a complicated and well-orchestrated physiological process in which blood clot formation is the initial and foremost phase to prevent excessive bleeding (Kolar et al., 2010; Claes et al., 2012). Beyond the hemostatic property, blood clots have proven to be critical to tissue healing, serving as a nature scaffold to deliver various growth factors and interact biologically with cells for tissue regeneration (Wang et al., 2017). Inspired by the natural healing blood clot, blood-derived products have attracted significant interests in recent years (Yang and Xiao, 2020).

Platelet-rich fibrin (PRF) prepared by centrifuging autologous blood is described as a natural fibrin scaffold containing all the constituents of blood that are favorable to tissue healing (Dohan Ehrenfest et al., 2012; Kumar and Shubhashini, 2013). For the past few years, PRF has been widely used in the treatment of bone deficiency in the field of stomatology, and has achieved remarkable curative effect owe to its fibrin network and growth factor release (Mazor et al., 2009; Oncu and Alaaddinoglu, 2015; Pradeep et al., 2015). However, freshly prepared PRF must be used immediately in order to retain the bioactivity of growth factors. In addition, like all natural scaffold materials, the bio-degradation rate of PRF is fast and irregular, along with the rapid release of growth factors, and then enzymatically hydrolyzed. Lyophilized PRF (L-PRF) is more conducive to preservation and transportation, that will contribute to its clinical application and promotion (Roseti et al., 2017). The pores of fibrin network in L-PRF are more abundant and larger, which would be in favour of cell migration and vascular invasion (Ma et al., 2018; Wang et al., 2019). What’s more, the freeze-thaw process further promotes platelet activation. However, accompanied by the change of network structure, the burst release of L-PRF after rehydration is more obvious (Roseti et al., 2017). Moreover, neither fresh nor lyophilized PRF has insufficient mechanical properties. Due to these deficiencies, their applications are mostly limited to combined use with other scaffold materials. In our previous study, we explored a controlled release strategy based on the combination of fresh and lyophilized PRF with different ratios, which increased bone formation *in vivo* (Liu et al., 2019). We consider that it is clinical significance to explore a simple and safe method to improve the performance of L-PRF for better applications.

Crosslinking has been a common method to improve the properties of biomaterials. Genipin (GP) extracted from gardenia jasminoides ellis fruit is a natural crosslinker with good biocompatibility and low cytotoxicity compared with other crosslinking agents such as glutaraldehyde (Frohbergh et al., 2012). GP can cross-link with amino acids, and the gardenia blue produced by the reaction is a safe, non-toxic natural food pigment with good anti-inflammatory effect. As one of the effective components of Chinese herbal medicine, genipin can be also used for anti-inflammatory, antibacterial, antithrombotic, anti-tumor drugs and treatment of diabetes, jaundice and other diseases (Koo et al., 2006; Hou et al., 2008; Lelono and Itoh, 2009). Therefore, in comparision with other chemical crosslinking agents, genipin is more biosafety, which eliminates the step of repeated rinse to remove the free crosslinking agent after crosslinking reaction is completed. In recent years, GP has been more and more applied in the field of tissue engineering, which can crosslink with a variety of natural scaffolds to improve the mechanical properties, regulate the degradation rate and control the release rate of drug (Yao et al., 2005; Yoo et al., 2011; Zhang et al., 2016). However, there is no study on appling GP to modify L-PRF yet. In our study, a variety of crosslinking schemes are designed to explore the feasibility of crosslinking L-PRF with genipin. We try to determin an simple and convenient crosslinking scheme and prepare a new type of GP crosslinked L-PRF scaffold. The physical and biological properties of the scaffold and the release kinetics of growth factors are investigated. The effect and application of genipin-modified L-PRF (GP/L-PRF) in bone regeneration are also explored.

## Materials and methods

### Preparation of L-PRF

Ten milliliters of autologous blood in 10 ml coated glass tubes without anticoagulants was obtained from the forearm vein of volunteers. The whole blood was immediately centrifuged at 3,000 rpm for 10 min (Labofuge 400Rcentrifuge, Heraeus, Hanau, Germany) according to the PRF protocol (Choukroun et al., 2006). Then the PRF clots were gently removed from the centrifuge tube, and the red blood cells at the bottom of PRF were cut off carefully. For the preparation of lyophilized PRF, PRF clots were frozen and stored at −80°C for 30 min and then freeze-dried overnight using a Labconco lyophilizer at −51∘C (Free Zone, Labconco, Kansas City, MO, United State).

### Crosslinking L-PRF with different concentrations of genipin

For each sample, 1 ml of GP was slowly added into L-PRF prepared from 10 ml of whole blood and mixed thoroughly. After 1 min, the excess solution was discarded. Then, samples were incubated for 24 h at room temperature to allow crosslinking reactions to proceed.

A series of gradient concentration GP solutions, including 0 w/v, 0.01% w/v, 0.1% w/v, 0.5% w/v and 1% w/v were applied to crosslink L-PRF. According to the concentration of genipin, the samples were grouped as 0 w/v L-PRF (non-crosslinked), 0.01% w/v GP/L-PRF, 0.1% w/v GP/L-PRF, 0.5% w/v GP/L-PRF and 1% w/v GP/L-PRF. All the experiments were performed in triplicates with three specimens in each group.

### Characterization of GP/L-PRF scaffold

Scaffolds of each group described above were then lyophilized again and 3 mg of the sample was weighed into EP tube respectively incubated with 100 μL of deionized water for 1 h. Then the ninhydrin (NHN) colorimetric assay was used to determine the degree of crosslinking as previously described (Sanchez et al., 2017).

Each lyophilized sample was weighed as *M*_*0*_ and then saturated with PBS (pH = 7.4) for 24 h at 37°C. The surface moisture was removed by blotting gently every half hour with filter paper and turgid weight *M*_*1*_ was recorded. Water absorption (*Wa*) was calculated as follows:

$$Wa=\frac{M1-M0}{M0}\times 100\%$$

The cross-sectional microstructure of scaffolds freeze-dried again was visualized using scanning electron microscopy (SEM; SM-5800LV, JEOL, Tokyo, Japan). The average pore diameter of crosslinked or non-crosslinked scaffold was calculated by ImageJ software.

The scaffold was cut into 2 mm thickness, from which standard disk sample of 2 mm diameter × 1 mm thickness was made using a tissue puncher. Mechanical properties were determined using a universal testing machine Intron5569.

### Quantification of growth factors derived from GP/L-PRF

First, extracts of all the groups of scaffolds were prepared. Briefly, either crosslinked or non-crosslinked lyophilized PRF scaffolds (0.1 g) were placed in 5 ml centrifuge tubes containing 1 ml of α-Minimum Essential Medium (α-MEM; Thermo Fisher Scientific, Inc. Waltham, MA, United States) without fetal bovine serum. The tubes were placed in a shaker incubator at 37°C at 100 rpm. The conditioned medium was collected at the times of 1, 3, 7, 14, 21, and 28 days of culture, and an equal volume of medium was added to the tubes. All collected mediums were stored at -80°C and analyzed at the same time to reduce bias. All ELISA kits were purchased from Boster Biological Technology and used according to the manufacturer’s protocol, and the wavelength for ELISA measurement was 450 nm. All assays were tested in triplicate.

### Cell culture

Culture of pulp stem cells from human exfoliated deciduous teeth (SHEDs) was carried out by adherent culture methods. Cells were cultured in α-MEM, supplemented with 10% fetal bovine serum (FBS; Thermo Fisher Scientific, Inc.), 100 U/mL penicillin and 100 mg/ml streptomycin (PS; Thermo Fisher Scientific, Inc.). They were incubated in a 5% CO_2_ atmosphere at 37°C.

To prepare cell membrane sheets, SHEDs were planted into 12-well plates with a density of 5  ×  10^5^ cells/mL. Cells were then grown continually without passaging for 14 days. Medium was changed every 48 h, and 50 mg/L ascorbic acid was added.

### Proliferation assay

0.1 g of scaffolds were placed into 24-well plates with 1 ml of complete α-MEM medium and incubated in incubator for 14 days. The conditioned medium was collected every day and an equal volume of medium was added. All collected media were stored at 4°C.

SHEDs were seeded at a density of 3,000 cells per well in 96-well plates. The incubation medium was discarded after 48 h culture, and the conditioned media mentioned above was added to each well following experimental grouping. Cell proliferation was measured by Cell Counting Kit-8 assay (CCK-8, Dojindo, Japan).

### Alizarin red staining and semi-quantitative analysis

To induce osteogenic differentiation, extracts were prepared as explained before, expecting that the α-MEM was replaced with osteogenic differentiation medium containing 10 nM of dexamethasone, 10 mM of β-glycerophosphate, and 100 μM of ascorbic acid (Sigma, Sigma Chemical Co., St. Louis, MO, United States).

SHEDs were seeded into 24-well cell culture plates at a concentration of 3 × 10^4^ cells/well. After cell attachment, different conditioned media were added to different wells following experimental grouping. After 14 days of co-culture, cells were fixed and stained using Alizarin Red S (ARS, Sigma, Sigma Chemical Co., St. Louis, MO, United States) for detecting mineralization. Then ARS staining was released by cetylpyridinium chloride (Sigma-Aldrich) and quantified by spectrophotometry at 560 nm.

### Quantitative PCR

Gene transcription and expression of RUNX2, COL-1 and OCN in SHEDs induced by conditioned media for 14 days were studied by qPCR analysis. Total RNA was extracted with the TRIzol reagent on the 14th day of osteogenic induction. QPCR was performed by an Mx3005P system using SYBR^®^ Premix Ex TaqTM (Takara Biotechnology Co., Ltd.), according to the manufacturer’s instructions. The RUNX2 primers were 5′-TGG​TTA​CTG​TCA​TGG​CGG​GTA-3′ (forward) and 5′-TCT​CAG​ATC​GTT​GAA​CCT​TGC​TA-3′ (reverse); the COL-1 primers were 5′- GAG​GGC​CAA​GAC​GAA​GAC​ATC -3′ (forward) and 5′-CAG​ATC​ACG​TCA​TCG​CAC​AAC-3′ (reverse); the OCN primers were 5′-CAC​TCC​TCG​CCC​TAT​TGG​C-3′ (forward) and 5′- CCCTCCTGCTTGGACACAAAG-3′(reverse); the β-actin primers were 5′-CAT​GTA​CGT​TGC​TAT​CCA​GGC-3′ (forward) and 5′-CTC​CTT​AAT​GTC​ACG​CAC​GAT-3′ (reverse).

### Animal surgical procedure

First, the implants were prepared as described below. Samples of each group were prepared in the form of circular discs of 5 mm diameter and 2 mm thickness with a 5 mm diameter skin biopsy puncher.

For *in vivo* degradation study, 1 cm long incisions were performed on the backs of rats with a distance of 2 cm from the midline, 2 cm intervals in the same side, and subcutaneous pockets created by blunt dissection. Scaffold samples were implanted in subcutaneous pockets on the backs of rats randomly. Animals were sacrificed after 1 week, 2 and 4 weeks of implantation. The implants were removed along with the surrounding skin and subcutaneous tissue, and perfused with 4% PFA immediately.

A total of 22 healthy male Sprague-Dawley (SD) rats, weighing around 200 g, were employed to construct the 5 mm critical calvarial bone defect model. After exposure of the cranium *via* skin incision, two 5 mm symmetrical full-thickness bone defects were created using an annular bone dill. The pre-prepared scaffolds and SHEDs membrane sheets were then implanted in the calvarial defects. The calvarial defects were randomly divided into seven groups: blank group (non-implant), Cell sheet (CS) group (only SHEDs membrane sheets), 0 w/v + CS group, 0.01% w/v + CS group, 0.1%w/v + CS group, 0.5% w/v + CS group and 1% w/v + CS group. The day of surgery was assigned as day 0. Rats were sacrificed with an overdose of 200 mg/ml pentobarbital sodium at 4 and 8 weeks after surgery. The entire cranium samples were extracted and stored in 4% paraformaldehyde for subsequent analysis.

### Plain X-ray radiography and micro-computed tomography

Radiographs of the cranium samples were taken by a Faxitron Specimen Radiography System (Model MX-20; Faxitron X-ray Corporation, Wheeling, IL) at 26 kVp and exposure time of 11 s. The degree of bone regeneration was examined by a micro-CT scanner (μCT35, Scanco Medical AG, Bassersdorf, Switzerland) with a 10 μm voxel size using the following parameters: 114 mA, 70 kVp, and exposure time of 300 ms. The new bone formation was calculated as the percentage fraction of new bone area to the total defect area using the methods described in previous study (Liu et al., 2019).

### Hematoxylin and eosin staining

After radiographic studies, the calvarial specimens were decalcified in 10% ethylenediaminetetracetic acid (EDTA) for about 4 weeks, sectioned by bisecting the 5 mm diameter defects, and then embedded in paraffin. Serial sections were prepared in the thickness of 4 μm from the middle part for hematoxylin and eosin staining (H&E).

### Statistical analyses

All statistical analyses were conducted using GraphPad Prism (v8.4.0). The data was statistically analyzed with one-way or two-way analysis of variance (ANOVA). All experiments were repeated at least three times, and all data were presented as mean ± standard deviation (SD). Differences with *p ≤ 0.05* were taken as statistically significant.

## Results

### L-PRF could crosslink with genipin

**FIGURE 1:**
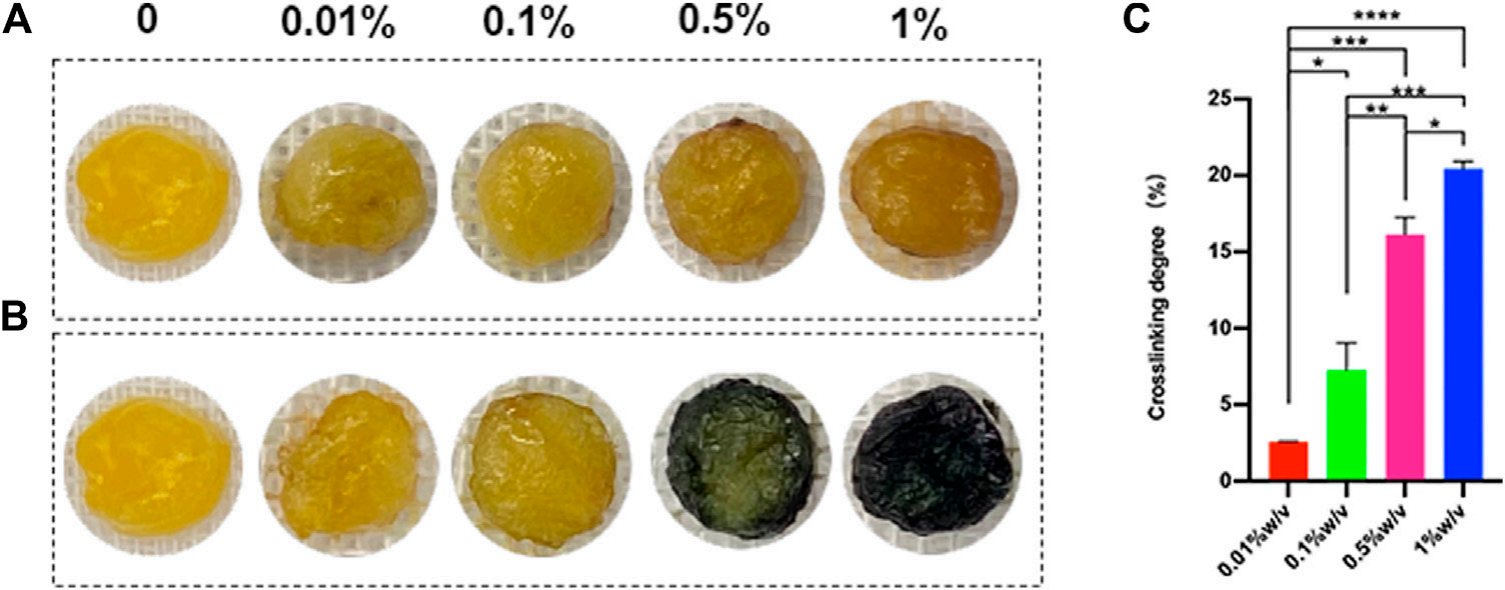
**(A)** Scaffolds crosslinked with genipin for 30 min **(B)** Scaffolds crosslinked with genipin for 24h, **(C)** The degree of crosslinking after 24 h (*p < 0.05, **p < 0.01,***p < 0.001,****p < 0.0001)

L-PRF could react with genipin to produce blue compound, and the blue color was deepened as time extended. The crosslinking time of 24 h was observed to be sufficient for complete crosslinking by prior experimentation. For the same duration of crosslinking, the greater the GP concentration used, the darker the blue color (Figures 1A,B). The ninhydrin (NHN) colorimetric assay was used to determine the degree of crosslinking. The result of the experiment shows the degree of crosslinking increased along with the increasing concentration of genipin (Figure 1C).

### Crosslinking improved the physical properties of L-PRF

**FIGURE 2:**
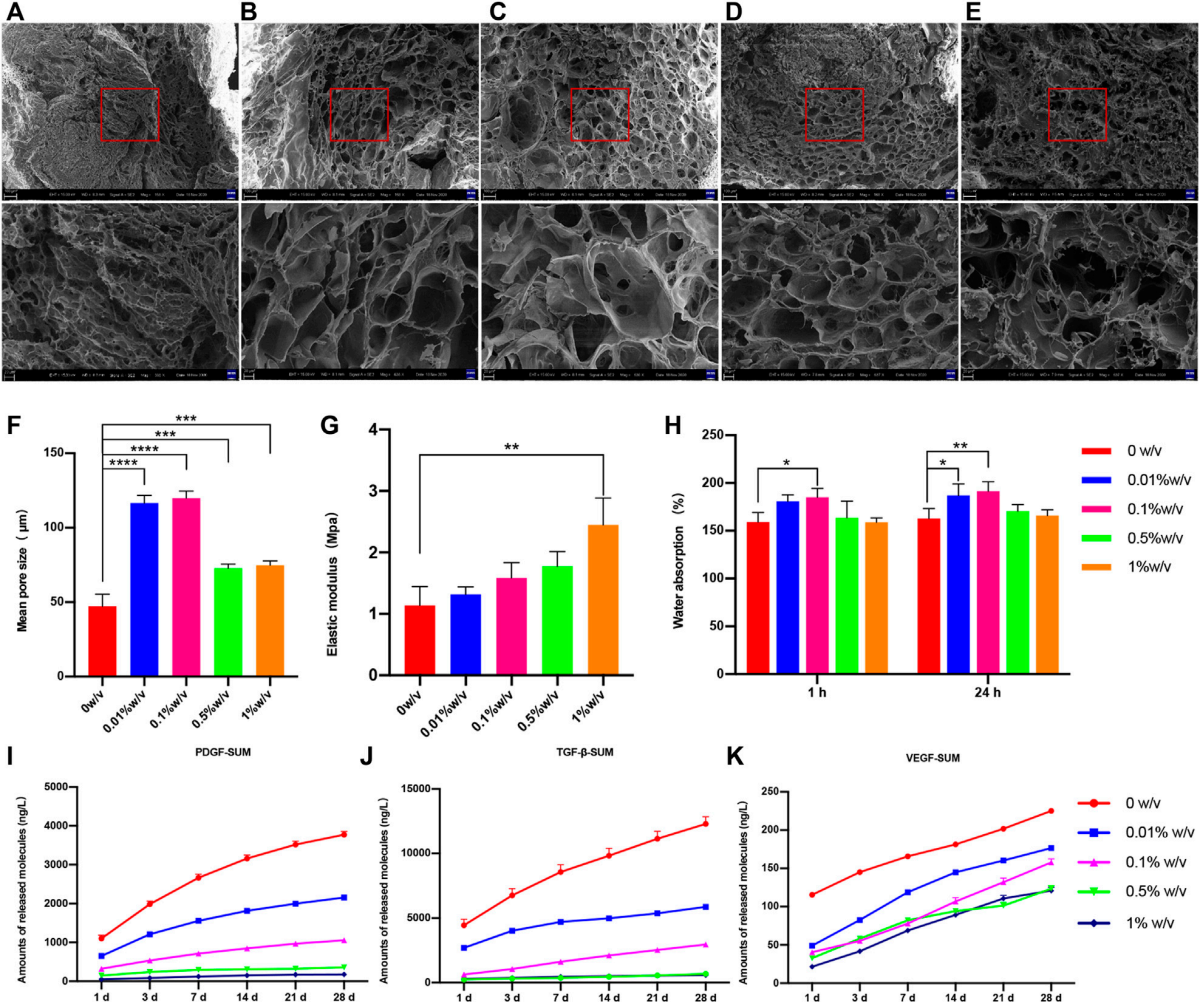
Scanning electron microscope (SEM) images of **(A)** 0w/v group **(B)** 0.01%w/v group **(C)** 0.1%w/v group **(D)** 0.5%w/v group and **(E)** 1%w/v group **(F)** The mean pore size of scaffolds, **(G)** Water absorption of scaffold **(H)** Elastic modulus of uncrosslinked and crosslinked group; **(I–K)** The cumulative release of growth factors of scaffolds. (The 0w/v group was used as control: *p < 0.05, **p < 0.01, ***p < 0.001, ****p < 0.0001, the release ofT GF-β1 at day1, 0.01%w/v VS control group, *p* > 0.05; other groups at all time points VS control group, *p* < 0.05).

Scanning electron microscopy image showed either crosslinked or non-crosslinked L-PRF scaffold had highly interconnected porous structures (Figures 2A–E). The number of macropores between 100–200 μm in diameter was increased in 0.01% w/v group and 0.1% w/v group compared to other groups, and adjacent macropores were interconnected by micropores.

The average pore diameter was measured from SEM images. The mean diameter was increased in each genipin crosslinked group compared with the non-crosslinked control group, and the differences were significant (Figure 2F). Of these, both 0.01% w/v GP/L-PRF and 0.1% w/v GP/L-PRF exhibited increasing average pore size larger than 100 μm (0.01% w/v vs control, and 0.1% w/v vs control, *p < 0.0001*).

The water absorption rates of the scaffolds were illustrated in Figure 2G. After 1 h immersion, the water absorption rate of all the samples tended to plateau, and reached the saturation limit after 24 h of immersion. Compared with the control group, all the GP crosslinked L-PRF showed increased water uptake, and 0.01% w/v GP/L-PRF and 0.1% w/v GP/L-PRF produced a significant difference.

The mechanical test results indicated that elastic modulus increased as the degree of crosslinking increased (Figure 2H). Elastic modulus of crosslinked L-PRF was higher than that of non-crosslinked L-PRF. The average modulus of elasticity of 1% w/v GP/L-PRF was significantly higher than control (*p < 0.05*). Although increasing values of elastic modulus were also shown in all other crosslinked groups, the differences were not significant compared with the control group.

### Crosslinking modified release kinetics of growth factors of L-PRF

The effect of genipin modification on the release kinetics of growth factors from L-PRF was studied by ELISA *in vitro*. The experiment was illustrated in Figures 2I–K and Supplementary Tables S1–S3. Although all groups had burst release within 24 h, the amount of GFs release from GP/L-PRF decreased with increasing crosslinking degree. After an initial burst release within the first day, GP/L-PRF showed slow release for the following days. The rate of GFs release decreased with the degree of crosslinking increased. The cumulative amount of PDGF-AB, TGF-β1 and VEGF in crosslinked groups were significantly lower than that in the control at all time points. The total release of three growth factors was different, with the highest release of TGF-β1, and the lowest release of VEGF.

### Crosslinking slowed the rate of degradation of L-PRF

**FIGURE 3:**
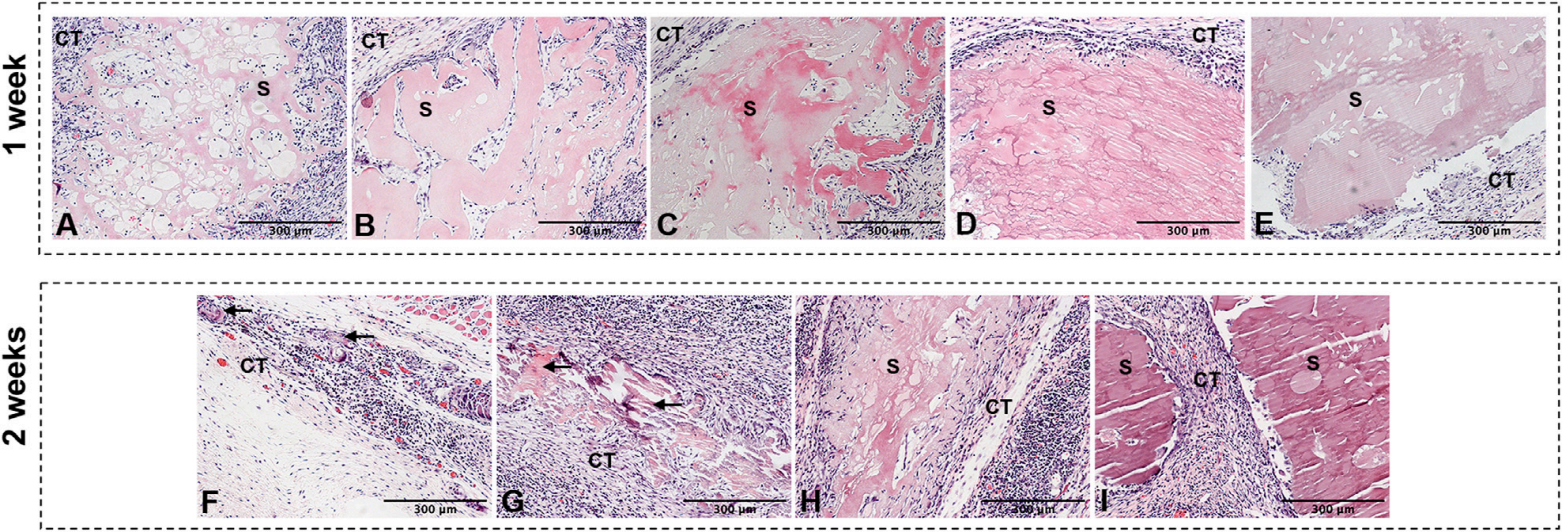
Photomicrographs of hematoxylin and eosin of **(A)** 0w/v group **(B)** 0.01%w/v group, **(C)** 0.1%w/v group **(D)** 0.5%w/v group, **(E)** 1%w/v group retrieved at 1 w and **(F)** 0.01%w/v group **(G)** 0.1%w/v group, **(H)** 0.5%w/v group **(I)** 1%w/v group retrieved at 2 w postoperatively. (S: scaffold, CT: connective tissue, Black triangular arrows indicate scattered scaffold debris).

The degradation of L-PRF was accompanied by the release of GFs. We then conducted subcutaneous implantation experiment to explore the biodegradability of GP/L-PRF *in vivo*, and observe its biocompatibility in the meanwhile. One week after surgery, the HE results showed that the scaffold material in each group was not completely biodegradable. Non-crosslinked L-PRF had a loose and thin fiber network, and contained a large number of infiltration cells (Figure 3A). With the crosslinking degree of L-PRF increased, the fiber network structure became increasingly compact, and the number of cells infiltrated within scaffolds markedly decreased. Scaffolds of 1% w/v GP/L-PRF showed compactness microstructure with few pores visible (Figures 3B–E).

The L-PRF of the non-crosslinked control group underwent complete biodegradation at 2 weeks after surgery (not shown). Only a small amount of scattered scaffold debris surrounded by inflammatory cells was presented in 0.01% w/v GP/L-PRF group (Figure 3F). Obvious degradation was also observed in the 0.1% w/v GP/L-PRF group. Most of the crosslinked L-PRF is replaced by fibrous connective tissue and cells. The boundaries between crosslinked scaffold and surrounding tissues were blurred, with new tissues growing inside the scaffold while the scaffold structure collapsed (Figure 3G). Many cells had infiltrated from the scaffold margin of 0.5% w/v group and fiber arrangement became looser (Figure 3H). There was no apparent degradation seen in 1% w/v group (Figure 3I). Inflammatory responses were not observed in any group. Abundant neovascularization was found around the scaffolds in all groups.

At 4 weeks, the scaffolds in all groups were completely degraded except 1% w/v group (Supplementary Figure S1B). Some granules with irregular shape could be seen in 0.5% w/v group (Supplementary Figure S1A). Neovascularisation was visible in the connective tissue.

As the results mentioned above, including average pore diameter and biodegradability showed the performance of 1% w/v GP/L-PRF was not satisfactory, the group was no longer shown in the following experiments.

### GP/L-PRF scaffold had No cell toxicity

**FIGURE 4:**
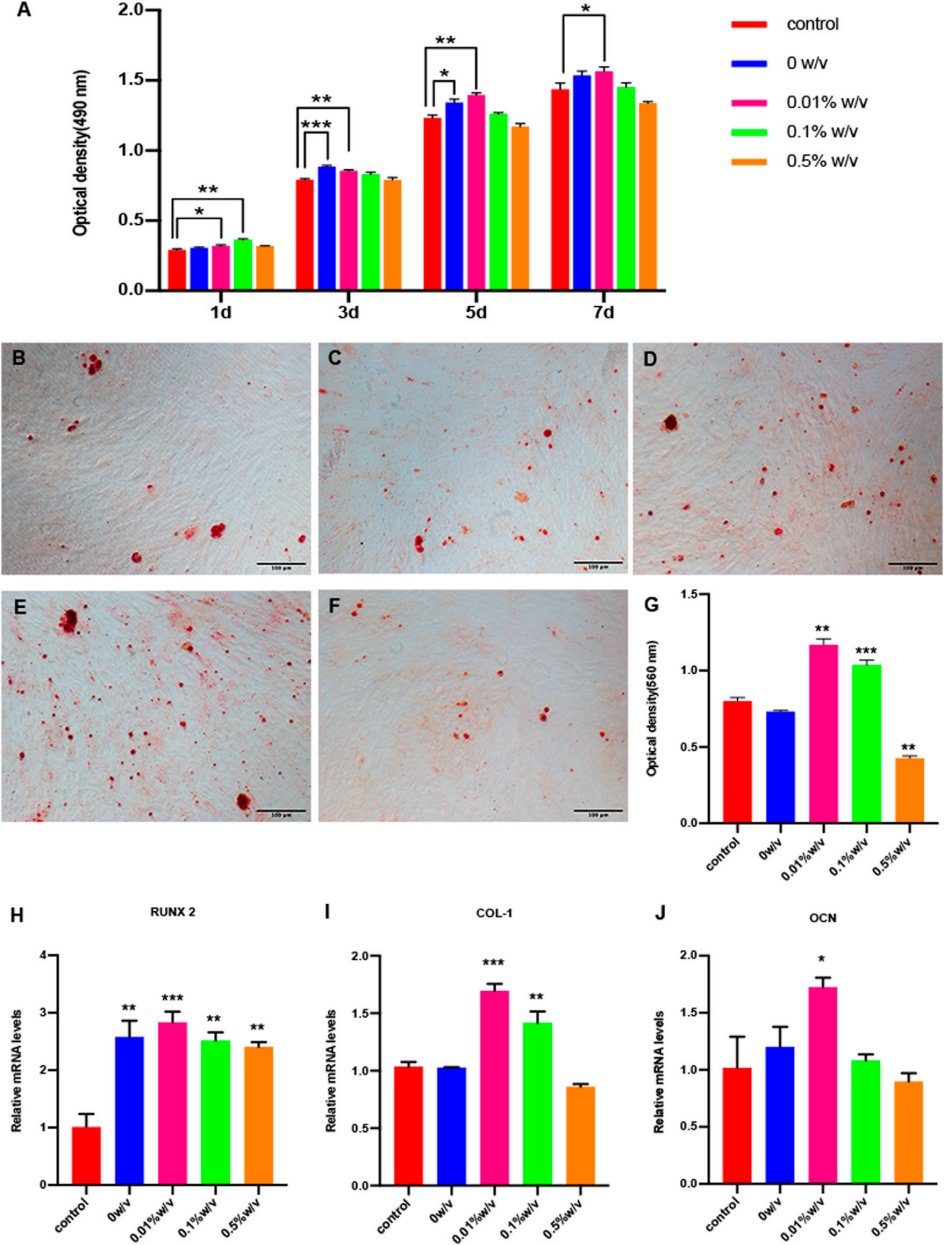
**(A)** Cell proliferation and cytotoxicity assay of SHEDs by CCK-8; Alizarin red staining of **(B)** Control group **(C)** 0w/v group **(D)** 0.01w/v group **(E)** 0.1%w/v group and **(F)** 0.5%w/v group **(G)** Semi quantitative results of alizarin red staining; **(H–J)** Real time PCR results of osteogenic–specific markers. (*p < 0.05, **p < 0.01, ***p < 0.001. RUNX2: Runt related transcription factor 2, COL-I: Collagen I,OCN: Osteocalcin).

To evaluate biocompatibility of crosslinked scaffolds, cytotoxicity test was performed on the extracts of scaffolds, and α-MEM complete medium was used as negative control group. As shown in Figure 4A, either non-crosslinked or crosslinked L-PRF showed no significant inhibitory effect on SHEDs proliferation at any of the time points examined. The extracts from 0.01% w/v GP/L-PRF could significantly promote the proliferation of SHEDs until day 7. The extracts from 0.5% w/v group reduced cell proliferation slightly.

### The extracts from GP/L-PRF enhance osteogenic gene expression

Mineralization nodules were detected through Alizarin red staining after 14 days of induction using an osteogenic differentiation medium (control) or corresponding extracts from 0w/v group, 0.01% w/v group, 0.1% w/v group and 0.5% w/v group. The mineralized nodules were more intense in the 0.01% w/v group and 0.1% w/v group, compared with those in the other groups. The 0.5% w/v group had the least mineralized nodule formation (Figures 4B–F). The result of semi-quantitative analysis of ARS showed that the OD values of 0.01% w/v group and 0.1% w/v group were significantly higher compared to the control group, at which the OD values of 0.01% w/v group were highest. 0w/v group and 0.5% w/v group represent no significant difference compared to control (Figure 4G).

The expression of osteogenesis related genes Runx2, Col-I and OCN were determined by qRT-PCR after 14 days of culture with osteogenic differentiation medium (control) or corresponding extracts from scaffolds. The 0.01% w/v group had the highest expression of all three genes compared to others. The expression of RUNX2 of cells with extracts treatment was higher compared with that of the control group (Figure 4H). COL-1 expression of 0w/v group and 0.5% w/v group did not show a significant expression difference when compared to the control, while the expression of 0.01% w/v group and 0.1% w/v group was significantly higher (Figure 4I). All groups except 0.5%w/v group significantly increased OCN expression on day 14 compared with control (Figure 4J).

### The 0.01% w/v and 0.1% w/v GP/L-PRF could promote new bone formation *in vivo*

**FIGURE 5:**
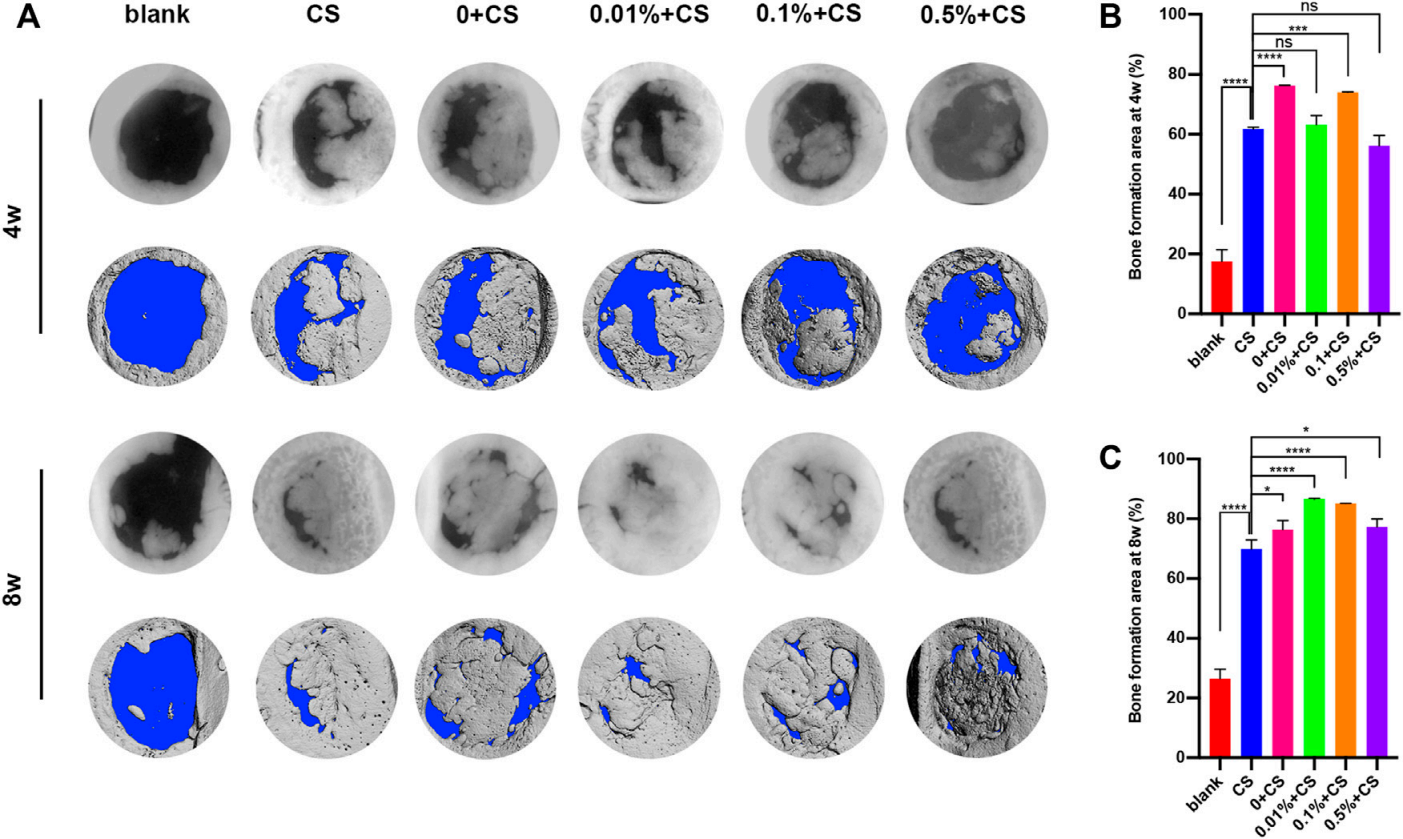
Radiographs and micro-CT analysis of bone regeneration in rat critical size calvarial defects at 4 and 8 weeks postoperatively **(A)** and quantification analysis of the regenerated tissue covering the calvarial defect at 4 w **(B)** and 8 w **(C)**. (The CS group was used as control: *p < 0.05, ***p < 0.001,****p < 0.0001. CS: cell sheet).

Finally, the *in vivo* repair efficacy of bone defects was evaluated in a rat defect model by implant GP/L-PRF combined with cell sheets. After implantation for 4 weeks, the blank group presented only a small amount of new bone tissues around the edge of defects. Various degrees of bone repair were observed on the edges and extended towards the center of the defect in all groups except blank control group (Figure 5A). Quantitative analysis of percentage of new bone area in calvarial defects revealed the area of newly formed bone in the 0w/v + CS group and 0.1% w/v + CS group were significantly greater than that in the CS group (Figure 5B).

After 8 weeks, the radiological findings showed an obvious increase in bone mass except for blank group. Continuous growth of new bone was still not observed in the blank group. The new bone of 0.01% w/v + CS group and 0.1% w/v + CS group filled almost the entire defect area and integrated with the original bone at the defect margins (Figure 5A). The percentage of new bone formation was (86.6 ± 0.2)% in 0.01% w/v + CS group and (85.1 ± 0.1)% in 0.1% w/v + CS group, significantly higher than that of the 0w/v + CS group. Compared with CS group, the area of newly formed bone tissues was significantly larger in each L-PRF combined with CS group (Figure 5C).

**FIGURE 6:**
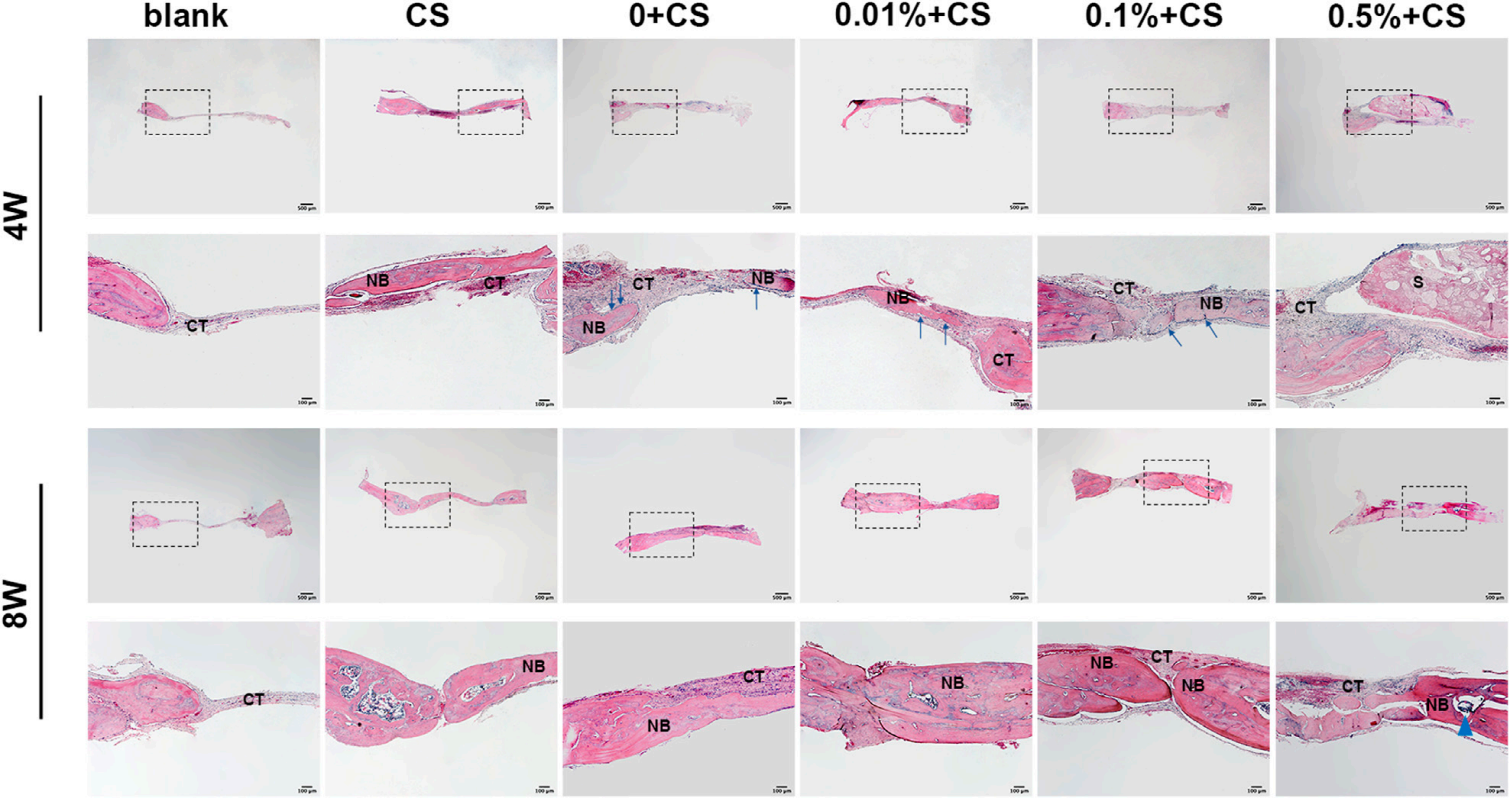
Photomicrographs of hematoxylin and eosin retrieved at 4 w and 8 w postoperatively. (S: scaffold, CT, connective tissue; NB, new bone; CS, cell sheet. The blue arrows indicate osteoblasts, Blue triangular arrows indicate the irregular particle residue).

The histological findings were consistent with imaging findings (Figure 6). In the blank control groups, almost no new bone was found in the defect 4 weeks postoperatively and only fibrous connective tissue covered the empty defects. A large area of residual materials could been seen in group of 0.5% w/v + CS. Histological sections of CS group, 0w/v + CS group, 0.01% w/v + CS group and 0.1% w/v + CS group presented abundant new island of bone and neovascularization. Osteoblasts were visibly arrayed on the surfaces of newly formed bone tissues in the 0w/v + CS group, 0.01% w/v + CS group and 0.1% w/v + CS group.

The defect area of the blank group still only had thin fibrous connective tissues at 8 weeks. Compared with 4 weeks, the trabecular bones were thickened, the number was increased and the connections were compact in CS group and scaffolds combined CS groups. The nascent bone in combination groups was thicker than that in CS alone group. It was obvious that trabecular bone in the 0.01% w/v + CS group and 0.1% w/v + CS group appeared to be thicker and more integrated, and the arrangement was more regular. The regenerated tissue completely fused with the host bone in the 0.01% w/v + CS group.

## Discussion

Blood clot plays a very important role during osteogenesis thanks to the structure characteristics and growth factors release (Lai et al., 2010; Shiu et al., 2018; Yang and Xiao, 2020). Inspired by the impact of natural blood clot, scholars have explored the innovation and application of blood products over the years (Yang and Xiao, 2020). Platelet-rich fibrin (PRF), a second generation of platelet concentrates, has already been widely used in modern medicine (Gassling et al., 2010; Sindel et al., 2017; Pripatnanont et al., 2013). Lyophilization was shown to activate resident platelets in PRF causing the further release of GFs thus promoting tissue healing (Roseti et al., 2017). However, the more obvious burst release of GFs of L-PRF cannot meet the requirement for bone tissue engineering (Li et al., 2014). In previous studies, we explored a biomimetic strategy to promote bone regeneration based on the combination of fresh and lyophilized PRF with different ratios (Sanchez et al., 2017). This study aims for tuning the release of GFs, decreasing the rate of degradation and improving the performance of L-PRF by crosslinking with genipin.

In order to accelerate bone reconstruction, an ideal implant requires both a porous structure and optimal water uptake properties, which is favorable for cell infiltration, adhesion and proliferation (Thavornyutikarn et al., 2014; Wang et al., 2016; Anitua et al., 2018). The average pore size shows a tendency to increase first and then decrease with the increase of the crosslinking degree. It could be observed that both macropores with a mean diameter larger than 100 μm and highly interconnected small pores coexisted in the 0.01% w/v group and 0.1% w/v group, meets the requirement for bone regeneration. The saturated water absorption of the 0.01% w/v group and 0.1% w/v group are significantly higher than that of non-crosslinked L-PRF, suggesting there is a higher porosity. The number of macropores is obviously reduced and the pore wall is nearly smooth almost without pores in the 1% w/v group, which is not adapted to bone reconstruction. After rehydration, non-crosslinked L-PRF lacks the required mechanical properties and maneuverability. As is shown in Figure 2G, the elastic modulus of non-crosslinked L-PRF is the lowest, whereas that of L-PRF crosslinked by 1% w/v genipin is the highest, illustrating crosslinking improves the mechanical properties of L-PRF.

Growth factors also play important roles in bone tissue engineering (Kneser et al., 2006; Iaquinta et al., 2019). PRF is naturally rich in growth factors (Dohan et al., 2006; Kumar et al., 2015). The soluble fibrinogen polymerizes slowly into fibrin network under the action of a physiological concentration of thrombin, during which growth factors are trapped in the fiber network (Dohan et al., 2006). Growth factors, which are not bound to fibrin, are rapidly released from PRF, while the bound growth factors are gradually released with the degradation of network (Atienza-Roca et al., 2018). Lyophilization promotes the further release of GFs derived from PRF, and meanwhile along with the change of network structure leads to a faster degradation and release rate (Li et al., 2014; Roseti et al., 2017). Previous studies have shown that the covalent bonding of GFs into scaffold can reduce the burst release (Masters, 2011). Muiznieks in his study stated that genipin could be used to crosslink elastin or elastin polypeptides by formed covalent crosslinks (Muiznieks, 2018). In this study, genipin is first used to crosslink with L-PRF. From the results, it can be seen that the burst release of GP/L-PRF is evidently decreased compared with L-PRF, suggesting the GFs may be conjugated onto fiber network which is necessary to further verify by further experiments. After burst release, GP/L-PRF releases GFs slowly and the cumulative release is always lower than non-crosslinked L-PRF during 28 days. The 0.1% w/v GP/L-PRF sustainably releases GFs at an almost constant rate. This slow-release effect also owes to the change of fibrin network structure. The structure characteristic of L-PRF includes a dense surface layer and loose inner structure. The surface pore architecture denser due to crosslinking is also contributed to controlled release.

The decreased rate of degradation is also in favor of the sustained release of GFs. The subcutaneous degradation experiment in rat shows that the higher the crosslinking degree is, the slower the degradation rate is. The non-crosslinked L-PRF completely degraded after 2 weeks of *in vivo* implantation, while 0.01% w/v and 0.1% w/v GP/L-PRF degraded to debris, 0.5% w/v GP/L-PRF degraded relatively slowly and 1% w/v GP/L-PRF showed no noticeable degradation. Hence, a significant amount of GFs would have been released at 1 week when referring to non-crosslinked L-PRF. The sustained release of 0.01% w/v and 0.1% w/v GP/L-PRF continues over a period of 2 weeks. The release rate is slower in 0.5% w/v GP/L-PRF and the slowest in 1% w/v GP/L-PRF. It can be found that the pattern of biomaterials degrading *in vivo* is in line with the pattern of GFs release *in vitro*. Degrading too slowly also has adverse effects of tending to be recognised as foreign and eliciting host immune responses against the antibody. It is suggested that the 1% w/v GP/L-PRF is not available for tissue healing. It is worth noting that in our subcutaneous degradation experiment, no obvious inflammatory reaction occurred in all groups, which may be attributed to the biocompatibility of blood derivatives, the modification of antigenic sites by crosslinking and lyophilization (Koo et al., 2006; Zhang et al., 2017; Yip et al., 2019). This may also be related to the anti-inflammatory effect of the crosslinked product geniposide, which needs further experiments.

Bone healing is regulated by various growth factors. It has been reported that bone repairing process involved revascularization, osteogenesis, bone remodeling, and the proliferation and differentiation of osteoblasts occurred during the initial 14 days (Uggeri et al., 2007). PDGF favors division and proliferation of osteoblasts within the damage site (Hollinger et al., 2008; Rao et al., 2009). TGF-β, the most abundant GFs derived from PRF, recruits circulated stem cells to the site of injury and promotes the deposition and mineralization of the bone matrix (Fennen et al., 2016; Perez et al., 2018). VEGF is a key component responsible for angiogenesis, which benefits stem cell recruitment and transportation of nutrients (Roseti et al., 2017; Iaquinta et al., 2019). Our research has shown that 0.01% w/v and 0.1% w/v GP/L-PRF can continuously release growth factors within 14 days. The *in vitro* studies find that the proliferation and osteogenic differentiation of SHEDs are stimulated by extracts derived from 0.01% w/v and 0.1% w/v GP/L-PRF. David *et al.* found PRF could both promote the proliferation of hBMSCs and enhance ALP activity and mineralized nodule formation. Sindel and others described that PRF enhanced the osteogenic ability of stem cell sheets. These findings are consistent with our studies. *In vivo* experiments in the present study also confirmed the new bone formation ratio of GP/L-PRF combined with SHEDs is higher than that of non-crosslinked L-PRF. By 8 weeks after the operation, 0.01% w/v and 0.1% w/v GP/L-PRF were able to almost completely heal the bony defect. The extracts of non-crosslinked L-PRF enhance the proliferative capability but not the osteogenic differentiation ability, that may be related to the high concentration of GFs. We find that new bone formation in 0.5% w/v GP/L-PRF group is slower because the GFs release is slower than other groups. From the above results, it is suggested that proper local concentration of GFs is important for bone tissue regeneration.

The cells cultured with conditioned medium from 0.5% w/v GP/L-PRF exhibited decreased proliferation from day 5, and decreased mineralized nodules and critical osteogenesis-related gene expression. We speculate the high concentration of genipin may be the reason. The dark blue particles observed in the *in vivo* experiment may provide evidence for excess GP.

This study is the first to present an approach to improve L-PRF by crosslinking with genipin. The present results show that reaction can be conducted at room temperature by very low concentrations of genipin. The obtained 0.01% w/v and 0.1% w/v GP/L-PRF has suitable pore structure and ability of sustained GFs release. Moreover, the crosslinked material presents good *in vitro* cell compatibility and *in vivo* biocompatibility. They can stimulate proliferation and osteogenic differentiation of SHEDs *in vitro* and increase bone formation on the bone defect area *in vivo*. GP/L-PRF combined with stem cell sheets accelerates bone defect healing. The present study provides a new way of thinking to improve the performance of L-PRF and an economical, safe and convenient method for sustained growth factors release.

## Data Availability

The raw data supporting the conclusion of this article will be made available by the authors, without undue reservation.
