# Supplementary material for: Genipin modified lyophilized platelet-rich fibrin scaffold for sustained release of growth factors to promote bone regeneration
Source: Front Physiol. 2022 Sep 30;13:1007692. doi: 10.3389/fphys.2022.1007692 (PMC9561255; doi:10.3389/fphys.2022.1007692)
Supplement: Supplementary file 1 [file DataSheet1.ZIP › Supplementary table revised.docx]

Supplementary Table 1 The levels of PDGF-AB released from non-croslinked or GP/crosslinked L-PRF

| Group | %w/v | Amounts of released molecules (mean and standard deviations) ng/L | | | | | |
| --- | --- | --- | --- | --- | --- | --- | --- |
|  |  | 1 d | 3 d | 7 d | 14 d | 21 d | 28 d |
| PDGF-AB | 0 | 1100.82 ± 83.59 | 885.59 ± 6.02 | 678.92 ± 23.58 | 494.94 ± 13.94 | 359.66 ± 6.63 | 252.30 ± 4.15 |
|  | 0.01 | 651.05 ± 15.19* | 560.29 ± 1  1.14** | 346.38 ± 9.57** | 257.57 ± 6.43*** | 181.72 ± 2.50*** | 159.88 ± 4.72*** |
|  | 0.1 | 320.84 ± 6.49** | 214.63 ± 0.79** | 177.24 ± 3.56** | 135.19 ± 5.96** | 120.05 ± 7.30*** | 86.24 ± 6.70*** |
|  | 0.5 | 149.188 ± 5.39** | 92.30 ±7.06** | 50.99 ± 4.19*** | 19.25 ± 0.68*** | 12.84 ± 2.32*** | 35.18 ± 1.54*** |
|  | 1 | 53.56 ± 3.97** | 30.59 ± 1.31** | 37.00 ±4.25*** | 30.62 ± 2.36*** | 20.62 ± 1.30*** | 6.18 ± 3.17*** |

Supplementary Table 2 The levels of TGF-β1 released from non-croslinked or GP/crosslinked L--PRF

| Group | %w/v | Amounts of released molecules (mean and standard deviations) ng/L | | | | | | |
| --- | --- | --- | --- | --- | --- | --- | --- | --- |
|  |  | 1 d | 3 d | 7 d | 14 d | 21 d | 28 d |  |
| TGF-β1 | 0 | 4439.29 ± 463.87 | 2314.29 ± 76.104 | 1812.65 ± 57.83 | 1269.56 ± 24.04 | 1312.46 ± 21.49 | 1139.33 ±27.82 |  |
|  | 0.01 | 2690.66 ± 152.04* | 1329.62 ±  23.74* | 679.75 ± 12.70* | 279.11 ± 13.03** | 394.36 ± 8.25** | 500.33 ± 4.40** |  |
|  | 0.1 | 634.68 ± 20.09* | 419.76 ± 5.19** | 560.58 ± 6.96** | 491.38 ± 4.47** | 429.61 ± 4.40** | 425.48 ± 57.50** |  |
|  | 0.5 | 243.80 ± 13.50** | 79.77 ± 7.67** | 44.00 ± 1.16** | 94.38 ± 10.00** | 95.82 ±9.62** | 123.82 ± 12.60** |  |
|  | 1 | 287.96 ± 10.70** | 80.58 ± 8.50** | 85.35 ± 8.27** | 49.24 ± 1.80** | 42.16 ± 3.14** | 38.65 ± 1.27** |  |

Supplementary Table 3 The levels of VEGF released from non-croslinked or GP/crosslinked L-PRF

| Group | %w/v | Amounts of released molecules (mean and standard deviations) ng/L | | | | | |
| --- | --- | --- | --- | --- | --- | --- | --- |
|  |  | 1 d | 3 d | 7 d | 14 d | 21 d | 28 d |
| VEGF | 0 | 115.50 ± 1.56 | 29.58 ± 0.75 | 20.76 ± 0.78 | 15.60 ± 0.89 | 20.39 ± 1.11 | 23.27 ± 0.32 |
|  | 0.01 | 49.15 ± 0.376*** | 33.44 ± 1.20**** | 36.21 ± 0.17**** | 26.00 ± 0.76*** | 15.43 ± 9.61** | 16.36 ± 0.56** |
|  | 0.1 | 40.47 ± 0.09*** | 14.74 ± 1.40**** | 22.79 ± 1.29**** | 28.86 ± 2.58*** | 25.11 ± 1.51** | 25.77 ± 1.51** |
|  | 0.5 | 32.75 ± 0.28*** | 25.56 ± 0.75**** | 23.83 ± 0.54**** | 12.07 ± 1.48**** | 7.13 ± 1.47*** | 22.12 ± 0.31*** |
|  | 1 | 21.55 ± 1.23**** | 20.13 ± 0.86**** | 27.15 ± 0.92**** | 20.48 ± 1.15*** | 21.49 ± 0.41*** | 10.09 ± 0.563*** |

Abbreviations: PDGF-AB, platelet-derived growth factor ; TGF-𝛽1, transforming growth factor-𝛽1; VEGF, vascular endothelial growth factor.

Statistically significant difference compared with 0w/v L-PRF group, **P<0.05*，***P<0.01, ***P<0.001*, *****p<0.0001*
