# Supplementary figures and images for: Genipin modified lyophilized platelet-rich fibrin scaffold for sustained release of growth factors to promote bone regeneration
Source: Front Physiol. 2022 Sep 30;13:1007692. doi: 10.3389/fphys.2022.1007692 (PMC9561255; doi:10.3389/fphys.2022.1007692)

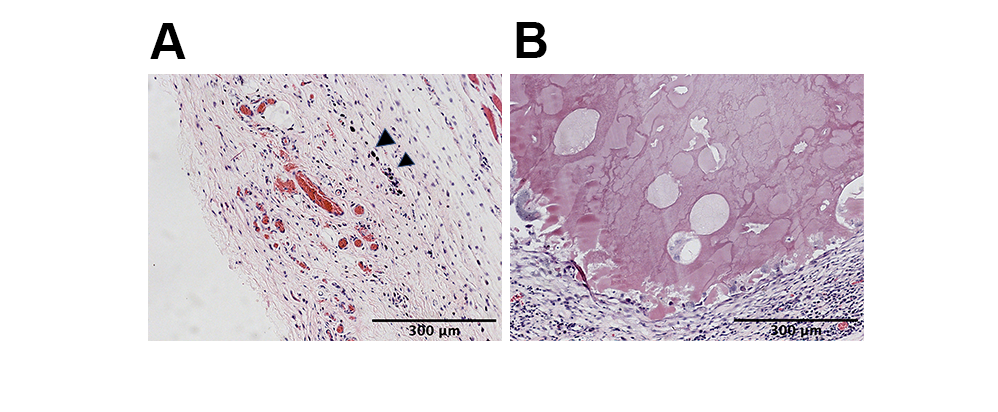

Supplement: Supplementary file 2 [file Image1.tif]
